# Molecular Variants in Human Trace Amine-Associated Receptors and Their Implications in Mental and Metabolic Disorders

**DOI:** 10.1007/s10571-019-00743-y

**Published:** 2019-10-23

**Authors:** Grazia Rutigliano, Riccardo Zucchi

**Affiliations:** 1 Institute of Life Sciences, Sant’Anna School of Advanced Studies, Piazza Martiri della Libertà 33, 56127 Pisa, Italy; 2 Institute of Clinical Physiology, National Research Council, Pisa, Italy; 3 Department of Pathology, University of Pisa, Pisa, Italy

**Keywords:** Trace amine-associated receptors, Genetics, Schizophrenia, Bipolar disorder, Single nucletide polymorphism

## Abstract

We provide a comprehensive review of the available evidence on the pathophysiological implications of genetic variants in the human trace amine-associated receptor (TAAR) superfamily. Genes coding for trace amine-associated receptors (*taars*) represent a multigene family of G-protein-coupled receptors, clustered to a small genomic region of 108 kb located in chromosome 6q23, which has been consistently identified by linkage analyses as a susceptibility locus for schizophrenia and affective disorders. Most TAARs are expressed in brain areas involved in emotions, reward and cognition. TAARs are activated by endogenous trace amines and thyronamines, and evidence for a modulatory action on other monaminergic systems has been reported. Therefore, linkage analyses were followed by fine mapping association studies in schizophrenia and affective disorders. However, none of these reports has received sufficient universal replication, so their status remains uncertain. Single nucleotide polymorphisms in *taars* have emerged as susceptibility loci from genome-wide association studies investigating migraine and brain development, but none of the detected variants reached the threshold for genome-wide significance. In the last decade, technological advances enabled single-gene or whole-exome sequencing, thus allowing the detection of rare genetic variants, which may have a greater impact on the risk of complex disorders. Using these approaches, several *taars* (especially *taar1*) variants have been detected in patients with mental and metabolic disorders, and in some cases, defective receptor function has been demonstrated in vitro. Finally, with the use of transcriptomic and peptidomic techniques, dysregulations of TAARs (especially TAAR6) have been identified in brain disorders characterized by cognitive impairment.

## Introduction

Trace amine-associated receptors (TAARs) belong to the family of G-protein-coupled receptors (GPCRs) (Zucchi et al. 2006; Grandy 2007). GPCRs, also known as seven-transmembrane receptors, represent the most versatile family of membrane receptors, as they respond to a broad range of stimuli, such as light, odorants, hormones, and several types of chemical messengers, including neurotransmitters (Pierce et al. 2002). The presence of a DRY motif (i.e. aspartate-arginine-tyrosine: unless otherwise specified, we are using single letter codes for amino acid residues) in the third transmembrane domain allocates TAARs to the largest class of GPCRs, known as Class A or Rhodopsin-like family (Borowsky 2001).

In mammals, the genes coding for TAARs (henceforward indicated as *taars*) are characterized by high homology, all cluster in a small region of a unique chromosome, with consistent transcriptional orientations across orthologs (Lindemann et al. 2005). All members of the *taar* family generate short (~ 1000-bp-long) intronless transcripts, with the exception of *taar2* which contains two exons. According to molecular evolutionary analyses, an ancestral gene emerged in the see lamprey (Eyun et al. 2016). Then, several species-specific events of gene duplications and pseudogenizations occurred, so that the number of *taars* is highly diverse among mammals, ranging from 0 in dolphins to 26 in the flying fox (Eyun et al. 2016). The receptors are classified into nine subfamilies (TAAR1 to TAAR9) (Hashiguchi and Nishida 2007). The oldest subfamily includes TAAR1, which is the only TAAR that is not expressed in the olfactory epithelium and does not function as an olfactory receptor (Eyun et al. 2016). Therefore, it appears that the divergence of younger TAARs from TAAR1 was accompanied by a change in their expression pattern (Eyun et al. 2016). From the functional point of view, receptors in the TAAR1-4 cluster detect primary amines, while those in the TAAR5-9 cluster, which are specific to therian mammals, are predominantly stimulated by tertiary amines (Ferrero et al. 2012).

**Fig. 1:**
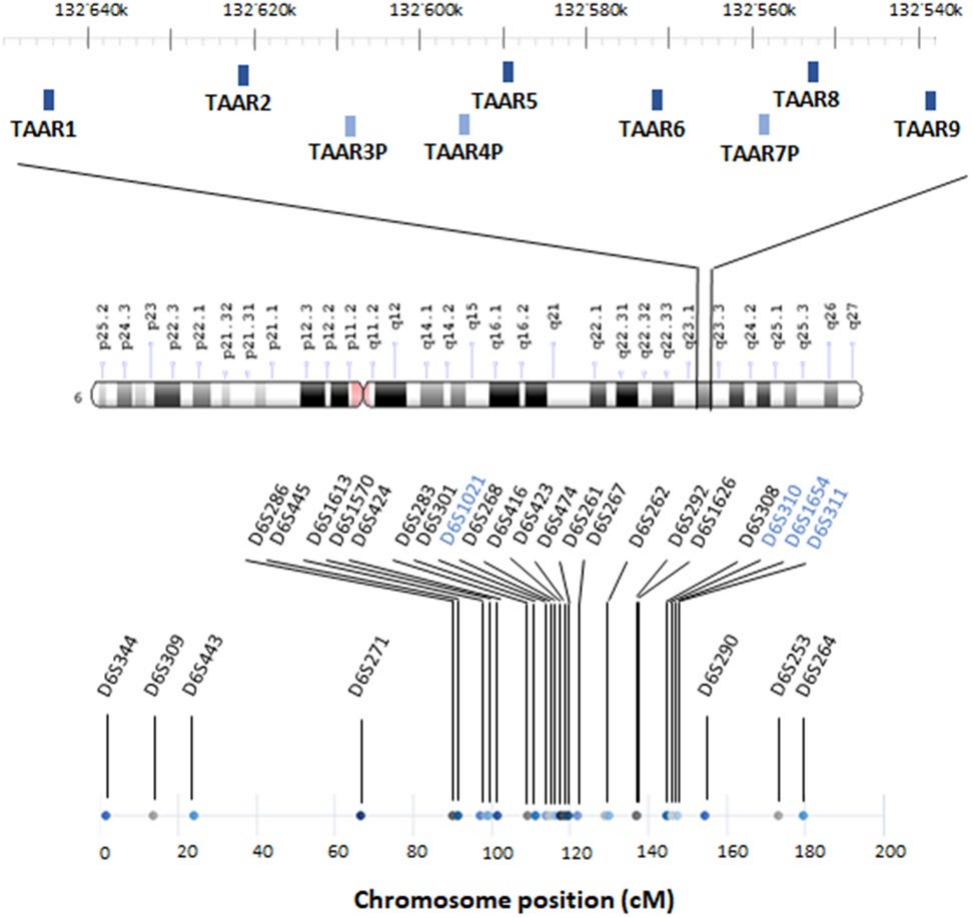
Schematic representation of the human 108-kb genomic region in chromosome 6q23, containing the genes coding for trace amine-associated receptors (*taars*). The location of the genes refers to the most recent Genome Reference Consortium assembly (February 2019), i.e. GRCh38.p13. The human genome encompasses six *taars* (*dark blue*) all present as single-copy genes. TAAR3, TAAR4, and TAAR7 (*light blue*) underwent pseudogenization before humans diverged from gorillas. All members of the *taar* family generate short (~ 1000-bp-long) intronless transcripts, with the exception of *taar2*, which contains two exons. This small genomic region has been repeatedly observed to be in genetic linkage with schizophrenia and bipolar disorder. Linkage studies pointed to a rather wide chromosomal region at 6q to contain one or more susceptibility loci for schizophrenia (~ 102–180 cM from the pter). The polymorphic markers showing the higher peaks in linkage with schizophrenia (*black*) or bipolar disorder (*blue*) are depicted in the lower part of the figure

While most genomes contain a single well-conserved copy of the more anciently emerged *taar* subfamily genes (*taar1*-*4*), the latest subfamilies (*taar5*-*9*) underwent multiple species-specific duplications, with positive selection, e.g. in *taar7* (Eyun et al. 2016). On the contrary, primate genomes are characterized by a small number of *taars*, with accelerated pseudogenization of *taar* repertoires (Eyun 2018). In particular, the human genome encompasses six *taars*, all present as single-copy genes, and mapping to a small genomic region of 108 kb located in chromosome 6q23 (Vladimirov et al. 2007). Functional TAAR3, TAAR4, and TAAR7 have been lost (Lindemann et al. 2005; Eyun et al. 2016), before humans diverged from gorillas (Fig. 1) (Staubert et al. 2010).

TAARs were identified while searching for novel biogenic amine receptors, but turned out to respond to trace amines, instead (Borowsky 2001; Bunzow 2001). The term trace amines refers to endogenous amines, namely β-phenylethylamine, p-tyramine, tryptamine, octopamine, and synephrine. They derive from aromatic amino acids and are physiologically present in tissues at much lower concentrations (< 100 ng/g tissue) (Boulton 1974) than the classic biogenic amines, such as dopamine, serotonin, norepinephrine, and histamine. While trace amines are major chemical messengers in invertebrates, in mammals they were originally believed to act as “false transmitters”, i.e. displacing classic biogenic amines from their stores and inhibiting their transporters (Parker and Cubeddu 1986). With the discovery of TAARs, it became clear that trace amines may exert actions in their own respect (Borowsky 2001; Berry 2004; Geracitano et al. 2004). Furthermore, some TAARs (notably TAAR1 and TAAR5) bind with high affinity to another class of endogenous amines, i.e. thyronamines, probably representing a novel branch of thyroid hormone signalling (Scanlan et al. 2004; Zucchi et al. 2014; Hoefig et al. 2016; Kohrle and Biebermann 2019). As a matter of fact, binding to trace amines has not been demonstrated for all TAAR subtypes, and this is the reason why the acronym TAAR has been accepted by the Human Genome Organization (HUGO) Gene Nomenclature Committee, although the International Union of Pharmacology (IUPHAR) still recommends the original denomination of trace amine receptors (Maguire et al. 2009).

As mentioned above, most TAARs are chiefly expressed in the olfactory epithelium and it has been argued that they might play a crucial role in sensing important ethological signals, such as predator and prey odours, spoiled food, migratory cues, and pheromones, thereby activating appropriate behaviours (Ferrero et al. 2011). For instance, β-phenylethylamine, which is ligand for TAAR1 and TAAR4, is a carnivore odour from mountain lions, tigers and jaguars (Ferrero et al. 2011; Dewan et al. 2013). *Taar* gene dynamics might be affected by environmental and evolutionary factors. Indeed, *taar* duplications and positive selection might have helped therian mammals to adapt to ground-living and discriminate among a wide number of volatile amines. On the other hand, *taars* deteriorated as a consequence of relaxed selection in those primates who adapted to arboreality, therefore relying less on olfaction for survival (Eyun 2018).

Notably, TAAR1 is also stimulated, with EC_50_ in the nanomolar to micromolar range, by a wide array of psychoactive drugs, such as amphetamine, 3,4-methylenedioxymethamphetamine, known as “ecstasy” (Bunzow 2001; Miller et al. 2005; Simmler et al. 2016), D-lysergic acid diethylamide, bromocriptine, lisuride, nomifensine, apomorphine, ractopamin, clonidine, guanabenz, idozoxan, aminoindanes (2-aminoindane and 5-iodo-2-aminoindane), and m-chlorophenylpiperazine (Bunzow 2001; Hu et al. 2009; Liu et al. 2014; Sukhanov et al. 2014; Simmler et al. 2016).

On the whole, it has been speculated that TAARs could play an important role in the central nervous system function, and possibly contribute an etiological role to the pathogenesis of mental disorders. This review will cover the efforts made, under a genetic perspective, to unravel the association between TAARs and psychopathology. The small genomic region on chromosome 6 where *taars* are located has been repeatedly observed to be in genetic linkage with schizophrenia and bipolar disorder. Fine mapping association studies were subsequently integrated with genome-wide association studies. Recent technological advances allowed large-scale sequencing of *taars*, as well as of the whole genomes of patients. Finally, the use of gene expression profiling and peptidome analyses has been used to identify TAAR dysregulation in specific diseases.

## Linkage Between Chromosome 6q23 and Mental Disorders

In linkage analysis, no assumptions about specific genes are made. Polymorphic markers scattered over the genome at approximately equal distances, i.e. microsatellites with di- or tri-nucleotide repeats, are genotyped in family members, to determine whether they segregated with the disease (Bray and O’Donovan 2019). Markers are usually indicated by symbols such as DXS000, where D stands for DNA, X is a number that identifies chromosomal assignment, S denotes that it is a unique DNA sequence, and the final number is a specific identifier (Fig. 1). Chromosomal location may be expressed with cytogenetic notation, where chromosome number is followed by the letter “p” (indicating the short arm) or “q” (indicating the long arm) and by a number that denotes microscope-identified bands and sub-bands. Distances between genes can be calculated on the basis of recombination frequencies, which are related to chromosomal crossover at meiosis and are expressed in centiMorgans (cM), where 1 cM corresponds to a frequency of 1% per generation. Distances can be also expressed as number of base-pairs calculated through sequencing, and in general in humans 1 cM corresponds to about one million base-pairs (1 Mbp). However, this is only an approximation, since recombination frequency is not the same in all regions.

The result of linkage analyses is expressed as logarithm of odds scores (LOD, i.e. logarithm to the base 10 of the likelihood of the observed data given linkage, divided by the likelihood of the data given no linkage), whose threshold for genome-wide significance is usually set at 3.3 (Lander and Kruglyak 1995). When dealing with complex traits, and/or constructing genetic maps, complex statistical methods are used, based on the so-called maximum likelihood estimates, and it is customary to use nonparametric linkage analyses (Risch 1990; Lander and Schork 1994).

Schizophrenia is a serious chronic mental disorder, with a prevalence of ~ 1% worldwide. Its pathophysiology apparently involves a complex genetic component, which accounts for about 80% of the heritability and interacts with environmental factors (Kety et al. 1994). Evidence suggesting linkage of chromosome 6q23 to schizophrenia dates back to 1997, when *Cao,* et al. showed: (i) in a first dataset of affected sibling pairs in 53 nuclear families, excess allele sharing for markers on 6q13-q26, with a peak close to marker D6S416 in the interval 6q21-q22.3 (*p* = 0.00024); (ii) in a replication dataset of 69 families from the NIMH Schizophrenia Genetics Initiative, maximum allele sharing at D6S424 (*p* = 0.0004), D6S283 (*p*= 0.0009) and close to D6S423 (*p* = 0.0009) (Cao et al. 1997). These results were confirmed by a follow-up study, performed by the same group in a replication sample including 43 pedigrees (54 independent sibling pairs) of American and Australian origin (Martinez et al. 1999). Further support derived from a multicentre, collaborative study, where 734 pedigrees (824 independent sibling pairs) from 8 samples were scanned with 8 markers on chromosome 6q (Levinson et al. 2000). Such positive findings were all delimited to a relatively centromeric region spreading from ~ 102 to 120 cM from the p terminal (pter). Another study reported significant linkage to schizophrenia in a more telomeric region at 6q25, with a peak at ~ 180 cM, in an ample Swedish pedigree (Lindholm et al. 2001). Similar locations were reported in 5 Austrian families (153.10 cM), and in 30 African-American pedigrees (138.11 cM) (Bailer et al. 2000; Kaufmann et al. 1998).

Besides the investigations focussing on specific chromosomal regions, a genome-wide survey of 71 families (86 independent sibling pairs, 462 markers) from Germany and Israel also found moderate to weak linkage signal in an area spanning an interval on 6q close to D6S271 (LOD = 1.12) and D6S1613 (LOD = 1.11) (Schwab et al. 2000). More robust linkage was described in the investigation in 21 Arab–Israeli families multiplex affected with schizophrenia (155 individuals) from the catchment area of a Regional Mental Health Center, a highly ethnically homogeneous population with high birth rate, peculiarly high level of consanguinity and a low rate of intermarriage with other population groups in Israel (Jaber et al. 1994, 2000). Isolated populations offer the advantage to lower the genetic complexity and the heterogeneity of the environmental background, thereby improving the chance to find susceptibility loci (Peltonen et al. 2000). Lerer et al. detected the strongest evidence for linkage at chromosome 6q23 at marker D6S292 (*p* = 0.000004), located 136.97 cM from the pter (Lerer et al. 2003). Taken altogether, these studies pointed to a rather wide chromosomal region at 6q to contain one or more susceptibility loci for schizophrenia (~ 102–180 cM from the pter). Therefore, Levi, et al. refined their analyses on the Arab–Israeli population. Such approach allowed them to strengthen the evidence of linkage at D6S1626 (136.97 cM), almost overlapping with the position reported in the previous genome scan, and to narrow the putative susceptibility region from 12.0 to 4.96 cM (Levi et al. 2005).

Of note, in the investigations of the Arab–Israeli population, three diagnostic models were applied, ranging from a narrow to a broad definition of schizophrenia. The most robust evidence was obtained under the broad diagnostic model, which also included nonaffective psychoses (Lerer et al. 2003; Levi et al. 2005). Interestingly, the same region of chromosome 6q has emerged as harbouring susceptibility loci for bipolar disorder as well. Bipolar disorder is a severely disabling chronic disorder, with an estimated prevalence of approximately 4% worldwide (Merikangas et al. 2011). It is a complex highly heritable disorder, whose onset is determined by an interaction between liability and protective genes, with environmental factors impacting the expression of such polygenic information (Escamilla and Zavala 2008). Using a high-density genome-wide linkage analysis of 25 extended multiplex Portuguese families, Middleton, et al. identified a significant evidence for linkage to bipolar disorder on chromosome 6q22 at position 125.8 Mb (LOD = 3.56) (Middleton et al. 2004). Similarly, the genome-wide scan of 153 pedigrees of bipolar families collected under the NIMH Genetics Initiative revealed a linkage at marker D6S311, located 147.5 cM from the pter (*p* = 0.008) (McInnis et al. 2003). The analysis of 250 independent pedigrees from the same population, confirmed the positive findings, but at a different location, namely at 114 cM, close to the marker D6S1021 (Dick et al. 2003). The strategy of performing linkage analyses in population isolates proved useful also in the context of bipolar disorder. In nine multigenerational families from the northern Swedish geographically isolated population of Västerbotten, one locus at chromosome 6q emerged as linked to bipolar disorder, with a LOD of 3.2 under the recessive model. This locus was located 146 cM from the pter in a candidate region between D6S310 and D6S1654 (Venken et al. 2005).

Notably, linkage studies are best suited for Mendelian disorders caused by one, or very few, genes, with a large impact on risk. Mental disorders generally adhere to complex patterns of inheritance, rather than to simple mono- or oligo-genic models, consistently with the difficulty in identifying risk loci and a clear mode of transmission in most of the affected families (Bray and O’Donovan 2019). Notwithstanding this limitation, linkage studies have provided precious hints on *taars* as possible candidate genes for mental disorders. Their results have been summarized in Fig. 1.

## Association Between *taars* and Mental Disorders

Linkage findings in chromosome 6q23 were followed by fine mapping association studies of *taars* (summarized in Table 1). Association studies are usually designed as case–control studies, in which a comparison is made of the frequency of genetic variants between affected and nonaffected individuals. Changes in single nucleotides are usually defined as single nucleotide polymorphisms (SNP) if they show population allele frequencies > 1%, and single nucleotide variants (SNV) if their frequency is < 1%. SNPs and SNVs are identified in the National Center for Biotechnology Information (NCBI) dbSNP database by an “rs” number, where “rs” stands for reference SNP cluster identification.

**Table 1:** Summary of the studies investigating molecular variants in human trace amine-associated receptors

References	Gene	Study design	Disease/phenotype	Sample size	Population
Duan et al. (2004)	*TAAR6*	Association study	Schizophrenia	827 subjects, from 192 families	European & African American
Abou Jamra et al. (2005)	*TAAR1* *TAAR5* *TAAR6*	Association study	Bipolar disorder	118 parent–offspring triads	European, German descent
				263 cases; 430 healthy controls	
Bly et al. (2005)	*TAAR2*	Gene sequencing	Schizophrenia	56 cases; 56 healthy controls	Caucasian
Ikeda et al. (2005)	*TAAR6*	Association study	Schizophrenia	405 cases; 401 healthy controls (*first*-*set analysis*)	Japanese
				503 cases; 440 healthy controls (*second*-*set analysis*)	
Amann et al. (2006)	*TAAR6*	Association study	Schizophrenia	85 affected; 34 parent–offspring triads; 19 families with ≥ 2 affected offspring	Arab–Israeli
Duan et al. (2006)	*TAAR6*	Association study	Schizophrenia	235 parent–offspring triads	Chinese Han
Venken et al. (2006)	*TAAR6*	Association study	Bipolar disorder	182 cases; 364 healthy controls; 9 families	Swedish^a^
Ludewick et al. (2008)	*TAAR6*	Association study	Schizophrenia	79 sib-pair families; 125 parent–offspring triads	European—German, Hungarian—and Israel
Pae et al. (2008b)	*TAAR6*	Association study	Schizophrenia Bipolar disorder MDD	281 schizophrenia; 190 bipolar disorder; 187 MDD; 288 healthy controls	Korean
Pae et al. (2008a)	*TAAR6*	Association study	Schizophrenia, PANSS scores	240 cases	Korean
Sanders et al. (2008)	*TAAR6*	Association study	Schizophrenia and schizoaffective disorder	1870 cases; 2002 healthy controls	European
Vladimirov et al. (2007)	*TAAR6* *TAAR2* *TAAR5*	Association study	Schizophrenia^b^	1408 subjects, from 265 families	Irish
Pae et al. (2009b)	*TAAR6*	Association study, epistasis with HSP-70	Schizophrenia	281 cases; 288 healthy controls	Korean
Pae et al. (2009a)	*TAAR6*	Association study, epistasis with HSP-70	Bipolar disorder; response to treatment	171 cases; 288 healthy controls	Korean
Vladimirov et al. (2009)	*TAAR6*	Association study	Schizophrenia or poor-outcome schizoaffective disorder	627 cases; 1021 healthy controls	Irish
Pae et al. (2010)	*TAAR6*	Association study	Response to antidepressant treatment; suicide	187 cases	Korean
Smith et al. (2012)	*TAAR1*	Association study	Fibromyalgia	496 cases; 348 healthy controls	Caucasian
Anttila et al. (2013)	*TAAR6 TAAR7P*	Genome-wide meta-analysis	Migraine	23 285 cases; 95 425 healthy controls (29 GWAS	European
Park et al. (2014)	*TAAR6*	GWAS	Change in %ΔFEV1 following ICS treatment	189 cases	Korean
Chang et al. (2015)	*TAAR6*	Association study	Change in %ΔFEV1 following ICS treatment	246 cases	Korean
John et al. (2017)	*TAAR1*	Exome sequencing	Schizophrenia	4 members from 1 family 475 + 310 cases; 410 healthy controls	North Indian (475) & American (310)
Jones et al. (2017)	*TAAR1* *TAAR2*	GWAS	Self-reported mosquito bite size	84 724 subjects	European
Mühlhaus et al. (2017)	*TAAR1*	Gene sequencing	Overweight/Obesity and disturbed glucose homeostasis	314 cases; 2018 healthy controls^c^	German
Shahin et al. (2018)	*TAAR2*	GWAS	Response to β‐blockers	699 subjects	Any
Szekely et al. (2018)	*TAAR8* *TAAR6*	GWAS	Cerebellar growth	LONG cohort: 458 subjects (119 with ADHD); Generation R: 257 subjects	Mixed^d^

*%ΔFEV1* percentage of forced expiratory volume in 1 s, *GWAS* genome-wide association study, *HSP*-*70* heat shock protein 70, *ICS* inhaled corticosteroids, *MDD* major depressive disorder

^a^The sample originates from the genetically isolated region of Västerbotten in northern Sweden

^b^Core schizophrenia, including schizophrenia, poor-outcome schizoaffective disorder and simple schizophrenia. No evidence of association for broader diagnostic categories: narrow psychosis spectrum, adding schizotypal personality disorder and all other nonaffective psychotic disorders; broad psychosis spectrum, adding mood-incongruent and mood-congruent psychotic affective illness, and paranoid, avoidant and schizoid personality disorders; very broad psychosis spectrum, including all psychosis spectrum disorders plus non-psychotic affective disorders, anxiety, alcoholism, etc

^c^Diabetes-free subjects older than 60 years of age from the population-based Study of Health in Pomerania (SHIP and SHIP-TREND) cohorts

^d^LONG cohort: 404 European Americans, 31 African Americans, 8 Asian Americans, 15 mixed ethnicity; Generation R: 177 White/Caucasian, 80 non-Caucasian (African, Asian, and Caribbean)

Significant case–control differences in allele or genotype distributions indicate either direct effects of the detected allele itself on liability to the condition, or linkage disequilibrium (LD) within the population between such detected (assayed) allele and the actual risk allele (Bray and O’Donovan 2019).

Most association studies have been focused on *taar6*, previously known as trace amine receptor 4. *Taar6* expression has been detected in the hippocampus, frontal cortex, amygdala and, at low levels, in the substantia nigra and basal ganglia (Borowsky 2001), all regions traditionally involved in the neurobiology of mental disorders (Grossberg 2000; Ivleva et al. 2017). *Taar6* was reported to be associated with schizophrenia in an ethnical heterogenous sample of 192 families, previously linked to 6q23. In detail, the most significant associated SNP was rs4305745, located in *taar6* 3′ untranslated region (3′-UTR), which could affect gene expression at the posttranscriptional level. This SNP exists in LD with two other rs6937506 and rs6903874, which, albeit associated, did not survive the Bonferroni correction for multiple testing (Duan et al. 2004). However, several studies failed to replicate these positive findings. As a matter of fact, no association between any of the assayed SNPs and schizophrenia was observed in Japanese (Ikeda et al. 2005) and European (Sanders et al. 2008) case–control samples, and families and parent–offspring triads of Arab–Israeli (Amann et al. 2006), Chinese Han (Duan et al. 2006), and European origin (Ludewick et al. 2008). Investigations aimed at detecting *Taar6* SNPs associated to bipolar disorder similarly led to negative results (Abou Jamra et al. 2005; Venken et al. 2006). Nonetheless, a case–control study in a Korean population confirmed the association of the SNP rs6903874 in the 3′-UTR with schizophrenia, and, additionally, bipolar disorder (Pae et al. 2008b). The same authors demonstrated that *taar6* SNPs might modulate the clinical presentation of disorders, in terms of symptom severity and response to treatment (Pae et al. 2008a, 2010).

Significant associations were also found in 265 pedigrees of the Irish Study of High-Density Schizophrenia Family between schizophrenia and the SNPs rs12189813 and rs9389011, and, more strongly, with two-four marker haplotypes including at least one of the former and/or SNP rs7772821. Notably, the strength of the association rose in subjects belonging to the core schizophrenia diagnostic groups and presenting with the most severe (top 20%) delusions and hallucinations (Vladimirov et al. 2007). The SNP rs7772821 is located in the 3′-UTR and was shown by in silico analyses to affect the secondary structure of the mRNA, with implications for mRNA folding, conformation, and stability, and hence gene expression. Also, sequences incorrectly folded might be available for pairing with miRNA, which interfere with translation. Indeed, in silico tools predicted four target sequences for four miRNAs, hsa-miR-92, hsa-miR-125a, hsa-miR-302b and hsa-miR-483, one of which (hsa-miR-125a) expressed in mouse brain (Lagos-Quintana et al. 2002). However, these findings could not be replicated in a case–control study performed by the same group in the same population (Vladimirov et al. 2009). Although this replication failure could be related to the different study design (multiplex families vs case–control), the possibility of false positive findings cannot be ruled out.

Apart from mental diseases, a few investigations evaluated the association between *taar6* SNPs and respiratory function indices, particularly the change in the percentage of forced expiratory volume in 1 s, following treatment with inhaled corticosteroids (see Table 1).

Table 1 also summarizes results related to the association of *taar1*, *taar2* and *taar5* SNPs with schizophrenia, bipolar disorder and fibromyalgia. In general, no significant relationship has been proven so far.

In conclusion, none of the reports of the association between *taars* and mental disorders has received sufficient universal replication, so their status remains uncertain. This lack of consistency can be explained by the small effects on susceptibility that are now known to typify common risk alleles, the low probability that any selected candidate allele is a true risk allele, and underpowered sample sizes (Bray and O’Donovan 2019). Quoting *Vladimirov,* et al. “If *taar6* does contribute to liability for schizophrenia, this contribution is relatively small and may vary significantly between samples” (Vladimirov et al. 2009).

## *Taar* Detection in Genome-Wide Association Studies

Over the last 12 years, technological advances in genotyping microarrays, together with the development of the HapMap project, made it possible to simultaneously genotype hundreds of thousands of SNPs in large samples. Such genome-wide association studies (GWAS) permitted to overcome the limitations of candidate gene approaches, such as: bias towards existing hypotheses, low probability of selecting a true risk allele from the millions present in the genome, low statistical power from small sample sizes. Therefore, they have now replaced the candidate/positional gene studies. Since common variants are related to small risk (odds ratios generally < 1.1), very large sample sizes are required to detect them at a significance threshold that controls for multiple (up to 1 million) testing, generally set at *p* < 5 × 10^−8^.

When interpreting GWAS, two main facts need to be taken into account: (1) most of the detected SNPs alter regulatory regions at a distance of several hundred kilobases from the gene of interest, rather than affecting the coding sequence; (2) linkage disequilibrium per se does not provide conclusive evidence about the causal role of a specific SNP *vs* the causal role of correlated SNPs (Bray and O’Donovan 2019).

At the time of writing, five large GWAS detecting *taar* genes have been published, whose findings are summarized in Table 1. SNPs in genes of the *taar* superfamily have emerged from GWAS investigating genetic variants associated with: migraine (*taar6*, *taar7p*) (Anttila et al. 2013); change in forced expiratory volume following treatment with inhaled corticosteroids (*taar6*) (Park et al. 2014); self-reported mosquito bite size (*taar1*, *taar2*) (Jones et al. 2017); response to β‐blockers (*taar2*) (Shahin et al. 2018); cerebellar growth (*taar6*, *taar8*) (Szekely et al. 2018). However, none of the detected variants reached the threshold for genome-wide significance.

## Copy Number Variants in *taar* Genes

Copy number variants (CNVs) are deletions, duplications or insertions larger than 1 kb present in human genome in multiple sites. These regions of copy number changes may vary in size between 1 kb and megabases, and account for 1.2% variation in an individual human genome, relative to the reference genome. It has been calculated that an individual human genome contains an average of 70 CNVs with a mean size of 341 kb (Redon et al. 2006). CNVs occur in the genome because of the presence of low copy repeats (LCRs), repetitive sequences of 10-500 kb with more than 90% sequence identity to another place in the genome. LCRs are substrates of non-allelic homologous recombination, leading to chromosomal rearrangement, namely deletion/insertion if the LCRs have the same orientation, and inversion, if LCRs are oppositely oriented (Rutkowski et al. 2017). CNVs are a major source of genome variability, which may be either benign or result in disease. Many factors play a role in determining the eventual impact of CNVs on human health, such as ethnical background, environment and other still unknown factors. At first, CNVs were identified with conventional karyotyping methods. Nowadays, much more sophisticated techniques are available, including array comparative genomic hybridization and, the most recent high-throughput next-generation sequencing. The clinical significance of CNVs must be interpreted carefully by geneticists, taking into account clinical information, such as parental inheritance, and considerations about CNV size (the smaller the size, the lower the risk of clinically relevant phenotypic consequences) and genomic content. Databases are available to guide CNV interpretation (Nowakowska 2017). Multiple studies have reported an enrichment of CNVs in neuropsychiatric disorders, including schizophrenia, autism spectrum disorders, intellectual disability, ADHD and Tourette syndrome, while CNVs seem to contribute less to disorders that are not commonly conceptualized as neurodevelopmental in origin, such as bipolar disorder and major depressive disorder (reviewed in (Rutkowski et al. 2017; Bray and O’Donovan 2019). Of note, such CNVs have some common features. First, beside neuropsychiatric disorders, they are associated with congenital craniofacial malformations. Second, they span large chromosomal regions, encompassing several genes with effects on several signalling pathways, cell types and tissues. Last, their contribution to risk is minimal, as compared to SNPs (Rutkowski et al. 2017). Similarly, CNVs have been described in association to metabolic disturbances as well, mainly diabetes (de Jesus Ascencio-Montiel et al. 2017). However, none of these analyses detected a significant association with the chromosomal region encompassing *taar* genes.

## Exome Sequencing and Changes in *taar* Gene Expression

It is now established that complex disorders, e.g. mental and metabolic disorders, are characterized by highly polygenic architectures, which, beside common variants of weak effect, involve rarer variants that potentially have a much greater impact on risk, and many pleiotropic genes (Smoller et al. 2019). The available SNP genotyping arrays are not able to capture such rare, often de novo, DNA variants (defined as single nucleotide variants, SNVs, as discussed above). Nevertheless, in the last decade, the massive developments in sequencing technologies has made possible to screen also for SNVs, either at the level of a single target gene or of the so-called exome, i.e. the part (~ 1%) of the genome that encodes proteins. Exonic mutations have the benefit to indicate specific genes, with predictable functional consequences, which makes them attractive for researchers seeking to generate cellular or animal models (Bray and O’Donovan 2019). An overall summary of the reported SNPs and SNVs in human *taars* is shown in Table 2.

**Table 2:** Single-nucleotide polymorphisms (SNPs) and variants (SNVs) in the human trace amine-associated receptor gene family

DbSNP database ID	Gene	Location (AA change)	Disease/phenotype	Association	References
rs4305745 (G>A)	*TAAR6*	3′-UTR	Schizophrenia	*p* = 0.0014	Duan et al. (2004)
				ns	Ikeda et al. (2005)
				ns	Duan et al. (2006)
				ns	Ludewick et al. (2008)
				ns	Vladimirov et al. (2007)
				ns	Vladimirov et al. (2009)
			PANSS scores	ns	Pae et al. (2008a)
			Response to antipsychotics	ns	Pae et al. (2009b)
			BD	ns	Venken et al. (2006)
			Schizophrenia; BD; MDD	ns	Pae et al. (2008b)
			Response to antidepressants; suicide	ns	Pae et al. (2010)
			BD; response to treatment	ns	Pae et al. (2009a)
rs6937506 (G>A)	*TAAR6*	3′-UTR	Schizophrenia	*p* = 0.0052, d.n.s. Bonferroni	Duan et al. (2004)
				ns	Ikeda et al. (2005)
				ns	Amann et al. (2006)
				ns	Duan et al. (2006)
				ns	Ludewick et al. (2008)
				ns	Vladimirov et al. (2007)
			BD	ns	Venken et al. (2006)
			Schizophrenia; BD; MDD	ns	Pae et al. (2008b)
			Suicide	*p* = 0.002	Pae et al. (2010)
			Response to antipsychotics	*p* < 0.001; balance accuracy = 0.53 (weak)	Pae et al. (2009b)
			BD; response to treatment	ns	Pae et al. (2009a)
rs6903874 (T>C)	*TAAR6*	3′-UTR	Schizophrenia	*p* = 0.0026, d.n.s. Bonferroni	Duan et al. (2004)
				ns	Ikeda et al. (2005)
				ns	Amann et al. (2006)
				ns	Duan et al. (2006)
				ns	Ludewick et al. (2008)
				*p* = 0.012	Pae et al. (2008b)
				ns	Vladimirov et al. (2007)
				ns	Vladimirov et al. (2009)
			PANSS scores	ns	Pae et al. (2008a)
			BD	ns	Venken et al. (2006)
				*p* = 0.005	Pae et al. (2008b)
			MDD, response to antidepressants	*p* = 0.006	Pae et al. (2010)
			Change in %ΔFEV1 following ICS	ns	Chang et al. (2015)
			Response to antipsychotics	*p* < 0.001; balance accuracy = 0.52–0.54 (weak)	Pae et al. (2009b)
			BD; response to treatment	ns	Pae et al. (2009a)
rs2840837 (A>G)	*TAAR6*	Intergenic	Schizophrenia	ns	Duan et al. (2004)
				ns	Amann et al. (2006)
rs1361280 (A>G)	*TAAR6*	Intergenic	Schizophrenia	ns	Duan et al. (2004)
				ns	Amann et al. (2006)
				ns	Vladimirov et al. (2007)
rs4473885 (C>T)	*TAAR6*	5′-UTR	Schizophrenia	ns	Duan et al. (2004)
				ns	Ikeda et al. (2005)
				ns	Amann et al. (2006)
rs4085406 (A>G)	*TAAR6*	5′-UTR	Schizophrenia	ns	Duan et al. (2004)
				ns	Ikeda et al. (2005)
				ns	Amann et al. (2006)
rs6907909 (A>G)	*TAAR6*	5′-UTR	Schizophrenia	ns	Duan et al. (2004)
				*p* = 0.0354 *(first*-*set analysis)* ns *(second*-*set analysis)*	Ikeda et al. (2005)
				ns	Amann et al. (2006)
				ns	Ludewick et al. (2008)
rs8192624 (G>A)	*TAAR6*	Exon, missense	Schizophrenia	ns	Duan et al. (2004)
				*p* = 0.014 *(family study)* *p* = 0.024 *(case*–*control)*	Abou Jamra et al. (2005)
				ns	Amann et al. (2006)
				ns	Vladimirov et al. (2007)
				ns	Vladimirov et al. (2009)
			Change in %ΔFEV1 following ICS	ns	Chang et al. (2015)
rs8192625 (G>A)	*TAAR6*	Exon, missense	Schizophrenia	ns	Duan et al. (2004)
				ns	Ikeda et al. (2005)
				ns	Amann et al. (2006)
				ns	Ludewick et al. (2008)
				ns	Sanders et al. (2008)
				ns	Vladimirov et al. (2007)
				ns	Vladimirov et al. (2009)
			PANSS scores	*p* = 0.04 *(total score)* *p* = 0.01 *(positive score)* *p* = 0.007 *(difference from baseline)*	Pae et al. (2008a)
			Response to antipsychotics	ns	Pae et al. (2009b)
			BD	ns	Abou Jamra et al. (2005)
				ns	Venken et al. (2006)
			BD; response to treatment	ns	Pae et al. (2009a)
			Schizophrenia; BD; MDD	ns	Pae et al. (2008b)
			Response to antidepressants; suicide	ns	Pae et al. (2010)
			Change in %ΔFEV1 following ICS	ns	Chang et al. (2015)
rs7772821 (T>G)	*TAAR6*	3′-UTR	Schizophrenia	ns	Duan et al. (2004)
				ns	Ikeda et al. (2005)
				ns	Amann et al. (2006)
				ns	Vladimirov et al. (2007)
				ns	Vladimirov et al. (2009)
			BD	ns	Abou Jamra et al. (2005)
			Change in %ΔFEV1 following ICS	*p* = 6.08 × 10^−5^, ns	Park et al. (2014)
			Change in %ΔFEV1 following ICS	*p*_corr_ = 0.002 *(codominant model)* *p*_corr_ = 0.03 *(dominant model)* *p*_corr_ = 0.01 *(recessive model)*	Chang et al. (2015)
rs7745308 (T>G)	*TAAR6*	3′-UTR	Schizophrenia	ns	Duan et al. (2004)
				ns	Amann et al. (2006)
rs6912930 (T>G)	*TAAR6*	3′-UTR	Schizophrenia	ns	Duan et al. (2004)
				ns	Amann et al. (2006)
			Change in %ΔFEV1 following ICS	ns	Chang et al. (2015)
rs7765655 (G>A)	*TAAR6*	Intergenic	Schizophrenia	ns	Duan et al. (2004)
				ns	Amann et al. (2006)
				ns	Vladimirov et al. (2007)
				ns	Vladimirov et al. (2009)
rs4129284 (C>T)	*TAAR6*	Intergenic	Schizophrenia	ns	Duan et al. (2004)
				ns	Amann et al. (2006)
rs9321354 (A>C)	*TAAR6*	3′-UTR	Schizophrenia	ns	Duan et al. (2004)
				ns	Amann et al. (2006)
rs9373026 (C>G)	*TAAR6*	5′-UTR	Schizophrenia	ns	Ikeda et al. (2005)
			Change in %ΔFEV1 following ICS	ns	Chang et al. (2015)
rs7452939 (G>A)^a^	*TAAR6*	3′-UTR	Schizophrenia	ns	Ikeda et al. (2005)
				*p* = 0.019, d.n.s. Bonferroni	Pae et al. (2008b)
				*p* < 0.001; balance accuracy = 0.57 (weak)	Pae et al. (2009b)
			PANSS scores	ns	Pae et al. (2008a)
			BD; response to treatment	ns	Pae et al. (2009a)
			Response to antidepressants; suicide	ns	Pae et al. (2010)
			Change in %ΔFEV1 following ICS	ns	Chang et al. (2015)
rs12189813 (G>C)	*TAAR6*	Intergenic	Schizophrenia	*p* = 0.02	Vladimirov et al. (2007)
				ns	Vladimirov et al. (2009)
rs9389011 (T>C)	*TAAR6*	Intergenic	Schizophrenia	*p* = 0.03	Vladimirov et al. (2007)
				ns	Vladimirov et al. (2009)
rs8192622 (C>T)	*TAAR6*		Schizophrenia	ns	Vladimirov et al.(2009)
rs3813354 (G>A)				ns	Vladimirov et al. (2007)
rs9389015 (C>T)				ns	
rs9389020 (T>C)	*TAAR6*		Schizophrenia	ns	Vladimirov et al. (2007)
				*p* = 0.0228	Vladimirov et al. (2009)
rs9493381 (G>T)	*TAAR6*	5′-UTR	Change in %ΔFEV1 following ICS	ns	Chang et al. (2015)
			Cerebellar growth	*p* = 1.27 × 10^−6^, ns	Szekely et al. (2018)
rs8192618 (G>A)	*TAAR1*	Exon, missense R23C)	Schizophrenia	NI—cases: 450/0/0^b^ US—cases: 296/4/0 controls: 401/0/0	John et al. (2017)
			Overweight/Obesity and disturbed glucose homeostasis	cases: 313/1/0^b^ controls: 2018/0/0	Muhlhaus et al. (2017)
rs8192620 (T>C)	*TAAR1*	Exon, synon (V288=)	BD	ns	Abou Jamra et al. (2005)
			Schizophrenia	NI—cases: 277/162/21^b^ US—cases: 170/75/9 controls: 230/139/31	John et al. (2017)
rs8192619 (G>A)	*TAAR1*	Exon, synon (C265=)	Fibromyalgia	*p* = 1.11 × 10^−5^	Smith et al. (2012)
			Schizophrenia	NI—cases: 394/66/0^b^ US—cases: 228/28/1 controls: 336/63/1	John et al. (2017)
ss28447860 (C>G)	*TAAR6*	Intergenic	Schizophrenia	ns	Duan et al. ((2004)
ss28447862 (T>C)				ns	
ss28447876 (G>A)				ns	
ss28447863 (A>G) ss28447865 (C>T)				ns	
ss28447866 (G>A)				ns	
ss28447871 (G>A)				ns	
rs8192627 (A>C)	*TAAR5*	Exon, missense	BD	ns	Abou Jamra et al. (2005)
Not reported	*TAAR2*	Exon, missense (W123STOP)	Schizophrenia	ns	Bly (2005)
rs17078770 (–/A)	*TAAR6*	3′-UTR	Schizophrenia	ns	Ikeda et al. (2005)
rs2842899 (T>A)	*TAAR6*	Intergenic	Schizophrenia	ns	Vladimirov et al. (2007)
rs2153174 (G>T)				ns	
rs1933988 (A>C)				ns	
rs2255071 (C>T)				ns	
rs7744878 (G>A)				ns	
rs7759367 (G>A)				ns	
rs17195227 (T>C)				ns	
rs17061477 (A>C)				ns	
rs4320395 (G>A)				ns	
rs9402439 (C>G)				ns	
Not shown	*TAAR2*	Not shown	Schizophrenia	ns	Vladimirov et al. (2007)
Not shown	*TAAR5*	Not shown	Schizophrenia	ns	Vladimirov et al. (2007)
rs9399032 (T>A)	*TAAR6; TAAR7P*	Intergenic	Migraine	*p* = 3.66 × 10^−6^, ns	Anttila et al. (2013)
rs11961899 (A>G)	*TAAR6*	5′-UTR	Change in %ΔFEV1 following ICS	ns	Chang et al. (2015)
rs9402427 (C>T)		5′-UTR		ns	
rs9402428 (G>A)		5′-UTR		ns	
rs9373024 (T>C)		5′-UTR		ns	
rs9389007 (A>G)		5′-UTR		ns	
rs11154685 (A>G)		3′-UTR		ns	
rs17802869 (T>G)		3′-UTR		ns	
rs367888752 (C>A)	*TAAR1*	Exon, missense (C182F)	Schizophrenia	Only present in affected individuals	John et al. (2017)
rs149785438 (G>C)		Exon, missense (S47C)			
rs567495315 (G>T)		Exon, missense (F51L)			
Chr6: 132966261 (G>C)^c^		Exon, missense (Y294T)			
Chr6: 132966259 (A>G)^c^		Exon, missense (L295S)			
rs182618382 (A>G)		Exon, synon (L253=)			
Chr6: 132966818 (C>T)^c^		Exon, missense (A109T)			
Chr6: 132966394 (A>G)^c^		Exon, missense (V250A)			
rs558642256 (T>A)		Exon, missense (S28C)			
rs73775159 (T>G)		Exon, synon (T37=)			
rs377606507 (C>T)		Exon, synon (E86=)			
rs77004036 (G>A)		Exon, synon (T102=)			
rs73557824 (A>G)		Exon, missense (Y173H)			
rs114701711 (A>G)		Exon, synon (L215=)			
rs540278394 (C>T)		Exon, missense (S244 N)			
rs6926857 (T>C)		Exon, missense (T252A)			
rs8192621 (T>C)		Exon, missense (R312R)			
rs184898731 (G>A)	*TAAR1; TAAR2*	Intergenic	Self-reported mosquito bite size	*p* = 8.64 × 10^−6^, ns	Jones et al. (2017)
rs140960896 (G>A) rs200795344 (T>A)	*TAAR1*	Exon, missense (S49L) Exon, missense (I171L)	Overweight/Obesity and disturbed glucose homeostasis	Cases: 313/1/0^b;^ controls: 2000/9/0^b^ Cases: 313/1/0^b;^ controls: not available	Muhlhaus et al. (2017)
rs9385619 (C>T)	*TAAR2*	5′-UTR	Response to β‐blockers	*p* = 8.23 × 10^−6^, ns	Shahin et al. (2018)

*%ΔFEV1* percentage of forced expiratory volume in 1 s, *BD* bipolar disorder, *d.n.s.* did not survive, *ICS* inhaled corticosteroids, *MDD* major depressive disorder, *NI* North Indian, *PANSS* positive and negative syndrome scale, *US* American, *UTR* untranslated region

^a^rs7452939 has merged into rs4305746

^b^WT/Hetero/variant homozygous genotypes

^c^Reference SNP cluster ID not available; reported as position in assembly GRCh37.p13

*Taar1* has been the preferential target of these investigations, because of its potential role in the pathogenesis of mental and metabolic disorders, which has been extensively reviewed elsewhere (Rutigliano et al. 2017; Gainetdinov et al. 2018; Schwartz et al. 2018). In summary, TAAR1 is expressed across brain areas involved in emotions, reward and cognition, where it may represent a rheostat of monoaminergic and glutamatergic neurotransmission. TAAR1 has also been detected in stomach, neuroendocrine cells of the intestine, β cells of the pancreas, and in brain areas involved in the control of energy metabolism. TAAR1 activation modifies nutrient-induced hormone secretion, and its potential role in diabetes and obesity is under investigation.

Striking evidence of the functional and behavioural consequences of spontaneous *taar1* mutations comes from genetic mouse models of voluntary methamphetamine (MA) drinking. By examining the progenitor strains of mouse lines selectively bred for high MA intake (MA drinking lines), it was found that the DBA/2 J strain bears a missense SNP in *taar1* at position 229, which could be traced as a spontaneous event at The Jackson Laboratory in 2001–2003. This SNP, which causes a proline to threonine amino acid change (*Taar*^*1m1J*^), leads to TAAR1 loss of function. The homozygous *Taar*^*1m1J*^ genotype is causally linked to increased MA consumption, reduced MA-induced conditioned taste aversion, and reduced MA-induced hypothermia (Reed et al. 2017).

In humans, a recent exome-sequencing study identified a heterozygous rare missense variant in *taar1* (rs367888752, C182F, Table 2) in the affected mother and two affected children but not in two unaffected siblings of a small schizophrenia family. This variant causes a cysteine to phenylalanine change in the second extracellular domain, thus breaking a highly conserved disulphide bond, which might tremendously impact on structure stability, cell surface localization, ligand binding and G-protein activation (Dohlman et al. 1990; Savarese et al. 1992; Perlman et al. 1995; Cook et al. 1996; Cook and Eidne 1997). Furthermore, the screening of *taar1* coding region yielded other seven rare variants (S47C, F51L, Y294T, L295S, L253 =, A109T, V250A; details provided in Table 2) in 785 patients affected with sporadic schizophrenia and none of the 410 healthy controls. Based on their finding of a significant enrichment in the burden of *taar1* rare variants (MAF < 0.001) among schizophrenia cases, the authors argued that *taar1* might be a strong schizophrenia risk-conferring gene (John et al. 2017). However, the potential functional impact of these rare variants was only assessed with in silico prediction tools.

*Taar1* has been screened for variants in a cohort of patients with impaired glycaemia and body weight regulation. Here, three heterozygous carriers of three rare missense variants were identified—R23C, S49L, and I171L (Table 2)—corresponding to a minor allele frequency (MAF) of 0.16%, which is substantially higher than in non-diabetic controls aged > 60 years in the general population-based Study of Health in Pomerania (Muhlhaus et al. 2017). The carrier of R23C showed complete loss of insulin production, while the other two carriers were obese/overweight patients with a slight impairment of glucose homeostasis. Accordingly, the functional in vitro characterization of the three variants revealed a complete loss of function for R23C, versus partial impairment in receptor signalling for S49L, and no effect for I171L. Strikingly, the carriers of the function-disturbing variants presented mental health issues, namely a borderline intellectual functioning (R23C), and some not otherwise specified “psychiatric problems” (S49L). Both involved aminoacid residues, namely Arg23 and Ser49, are highly conserved among TAAR1 orthologues. Arg23 is located at the transition between the N-terminal tail and the transmembrane helix 1, where its side chain might interact with negatively charged residues of the extracellular loop 1 (Glu86 or Asp21). Ser49 points to the cytosol at the transition between the transmembrane helix 1 and the intracellular loop 1, and may contribute to the maintenance of the receptor structural conformation and to G-protein activation. Therefore, a role for *taar1* variants in the pathophysiology of insulin secretion, glucose homeostasis, and weight regulation could be speculated.

The functional consequences of the ~ 50 *taar1* variants reported in the dbSNP database still awaits clarification. In 2016, Shi et al. identified eight variants located in highly conserved motifs, to be characterized for agonist-induced function (C74Y, C182Y, K218I, T252A, C263G, C265 W, W291STOP, N300 K, R312S). Upon stimulation with β-phenylethylamine, no cAMP increase was observed in cells transfected with C74Y, T252A, and C265 W, while a sub-optimal cAMP response was observed for C182Y and K218I (Shi et al. 2016).

Finally, a study with an underpowered sample size described a SNP of *taar2*, which replaces a tryptophan at position 123 with a stop codon. This SNP seems to be slightly more frequent in patients with schizophrenia, as compared to healthy controls, although statistical significance was borderline. Moreover, there is still no evidence of *taar2* expression in the brain, so the relevance of this finding is uncertain (Bly 2005).

It is possible that carriers of sub-functional receptors might benefit from treatments with TAAR1 agonists. The current status of the development of TAAR1 agonists as therapeutic agents for mental and metabolic disorders has been recently summarized in (Berry et al. 2017). Briefly, preclinical evidence indicates that TAAR1 agonists might be useful for the treatment of schizophrenia. Indeed, TAAR1 agonists appear to offer some interesting advantages as compared to commonly used antipsychotics. Beside controlling hyper-dopaminergia, classically considered the neurochemical underpinning of positive symptoms of schizophrenia, they act on hypo-glutamatergia, possibly related to negative and cognitive schizophrenia symptoms. Also, they do so while limiting common side effects of antipsychotics, such as extra-pyramidal side effects and olanzapine-induced weight gain. As such, a mixed 5HT1A/TAAR1 agonist, SEP-363856 (Sunovion Pharmaceuticals), is under phase I clinical trial for schizophrenia, with promising results. Interestingly, SEP-363856 compound was shown to modulate the dopamine reward pathway, according to the preclinical observation of anti-craving effects of TAAR1 agonists for drugs that activate the dopaminergic reward pathway. As regards metabolic disturbances, clinical testing has not begun as yet, although TAAR1 agonists demonstrated, in relevant animal models, to induce weight loss, improve insulin sensitivity, reduce plasma and hepatic triglycerides.

Because of the variability in penetrance, genome sequencing should be associated to profiling gene expression and peptide regulation. The term gene-expression profiling refers to the simultaneous assessment of the relative transcript abundance of a large number of genes, in multiple experiments comparing different conditions. According to the scope, mRNA levels are assayed with microarrays or next-generation sequencing technologies (RNA-seq). Similarly, the peptidome assessment refers to the investigation of the array of endogenous peptides present in the intracellular and extracellular space of the body in a system-wide manner and holds the promise to facilitate the identification of biomarkers.

Using gene expression profiling, differential expression of 721 genes was demonstrated in the hippocampus in an animal model of autism, consisting of valproic acid-treated rats. In particular, *taar7* *h* and *taar7b* emerged as hub genes in the significantly downregulated neuroactive ligand-receptor interaction pathway. These findings suggest that TAARs might play an important role in autism spectrum disorders (Huang et al. 2016). Accordingly, the peptidome analysis of 68 Han Chinese children with autism and 80 healthy controls allowed the identification of 8 potential serum biomarker peaks, including TAAR6 (Yang et al. 2018). A significantly different peak area of the TAAR6 fragment was also reported in 30 patients with vascular cognitive impairment, relative to 30 healthy controls (Xi et al. 2009).

However, the direction of the differences was opposite, in that TAAR6 levels were elevated in autism spectrum disorders, and reduced in vascular cognitive impairment. Therefore, these results should be interpreted with great caution, and validated in independent samples.

## Conclusions

On the basis of the expression, pharmacology, and putative functional role of TAARs, it was hypothesized that subjects carrying mutant receptors might be predisposed to several diseases, particularly mental and metabolic disorders. Following the evidence of a linkage between chromosome 6q23 and schizophrenia or affective disorders, several teams have attempted to find susceptibility loci for mental disorders in *taars*, especially *taar6*, with inconclusive results. Similarly, susceptibility loci identified close to *taars* by GWAS did not reach genome-wide significance. However, rare variants have been detected in *taars*, particularly in *taar1*, by sequencing screening in cohorts of patients with mental and metabolic disorders. Such rare variants were demonstrated in vitro to disrupt the function of the receptor. It is possible that carriers of these nonfunctional variants could be at higher risk for mental and metabolic disorders, and individuals carrying sub-functional receptors might benefit from treatments with TAAR1 agonists. Finally, results of transcriptomic and peptidomic analyses, although very preliminary, suggest that TAARs, in particular TAAR6, might be involved in the pathophysiology of autism spectrum disorders and vascular cognitive impairment. In conclusion, some members of the human TAAR superfamily could represent promising targets for the treatment of mental and/or metabolic disorders, but further investigation is needed to reach definite conclusions.
